# The sero-epidemiology of Rift Valley fever in people in the Lake Victoria Basin of western Kenya

**DOI:** 10.1371/journal.pntd.0005731

**Published:** 2017-07-07

**Authors:** Elizabeth Anne Jessie Cook, Elysse Noel Grossi-Soyster, William Anson de Glanville, Lian Francesca Thomas, Samuel Kariuki, Barend Mark de Clare Bronsvoort, Claire Njeri Wamae, Angelle Desiree LaBeaud, Eric Maurice Fèvre

**Affiliations:** 1 Institute for Immunology and Infection Research, University of Edinburgh, Edinburgh, United Kingdom; 2 International Livestock Research Institute, Nairobi, Kenya; 3 Department of Pediatrics, Stanford University School of Medicine, Stanford, United States of America; 4 Centre for Microbiology Research, Kenya Medical Research Institute, Nairobi, Kenya; 5 The Roslin Institute, University of Edinburgh, Roslin, United Kingdom; 6 Royal (Dick) School of Veterinary Studies, University of Edinburgh, Roslin, United Kingdom; 7 Department of Microbiology, Mount Kenya University, Thika, Kenya; 8 Institute of Infection and Global Health, University of Liverpool, Neston, United Kingdom; CDC, UNITED STATES

## Abstract

Rift Valley fever virus (RVFV) is a zoonotic arbovirus affecting livestock and people. This study was conducted in western Kenya where RVFV outbreaks have not previously been reported. The aims were to document the seroprevalence and risk factors for RVFV antibodies in a community-based sample from western Kenya and compare this with slaughterhouse workers in the same region who are considered a high-risk group for RVFV exposure. The study was conducted in western Kenya between July 2010 and November 2012. Individuals were recruited from randomly selected homesteads and a census of slaughterhouses. Structured questionnaire tools were used to collect information on demographic data, health, and risk factors for zoonotic disease exposure. Indirect ELISA on serum samples determined seropositivity to RVFV. Risk factor analysis for RVFV seropositivity was conducted using multi-level logistic regression. A total of 1861 individuals were sampled in 384 homesteads. The seroprevalence of RVFV in the community was 0.8% (95% CI 0.5–1.3). The variables significantly associated with RVFV seropositivity in the community were increasing age (OR 1.2; 95% CI 1.1–1.4, p<0.001), and slaughtering cattle at the homestead (OR 3.3; 95% CI 1.0–10.5, p = 0.047). A total of 553 slaughterhouse workers were sampled in 84 ruminant slaughterhouses. The seroprevalence of RVFV in slaughterhouse workers was 2.5% (95% CI 1.5–4.2). Being the slaughterman, the person who cuts the animal’s throat (OR 3.5; 95% CI 1.0–12.1, p = 0.047), was significantly associated with RVFV seropositivity. This study investigated and compared the epidemiology of RVFV between community members and slaughterhouse workers in western Kenya. The data demonstrate that slaughtering animals is a risk factor for RVFV seropositivity and that slaughterhouse workers are a high-risk group for RVFV seropositivity in this environment. These risk factors have been previously reported in other studies providing further evidence for RVFV circulation in western Kenya.

## Author summary

Rift Valley fever virus (RVFV) is a zoonotic virus affecting livestock and people. Periodic outbreaks in Kenya are associated with greater than average rainfall, although outbreaks have not previously been reported in western Kenya. The virus is spread between animals and to people by mosquitos. Contact with infected animal tissues and products are also risk factors for transmission of RVFV to people. This study investigated the seroprevalence of RVFV in 1861 residents of western Kenya and compared this to the seroprevalence in 553 ruminant slaughterhouse workers. The seroprevalence of RVFV in people in western Kenya was less than 1%, which is consistent with previous reports from the region. Slaughterhouse workers were shown to be a higher risk group for RVFV seropositivity, with seroprevalence of 2.5%. The identification of plausible risk factors including slaughtering is consistent with reports from other regions. The results suggest that it is plausible that RVFV virus is circulating in western Kenya. Improved surveillance in low risk areas is recommended particularly during countrywide outbreaks.

## Introduction

Rift Valley fever virus (RVFV) is a zoonotic arbovirus affecting livestock and people in Africa and the Arabian peninsula [1]. Epidemics of Rift Valley fever (RVF) are associated with greater than average rainfall, and are characterised by abortion in livestock and febrile illness in people [1,2]. RVFV outbreaks have not previously been reported in western Kenya since the initial discovery of the virus in the Rift Valley in 1931, although epidemics have occurred in neighboring regions [3]. It has been suggested that the virus can be maintained in animal populations between epidemics and potentially spread to new areas through animal movement [4]. Previous work has documented low-levels of RVFV exposure in western Kenya, compared to high-levels in north-eastern populations, but exposure to RVFV in high-risk occupations in western Kenya has not been examined [4,5]. The climate of western Kenya is sub-tropical with consistently high temperatures and humidity, and predictable rain/dry season cycles. The study area is semi-humid to humid with greater than 1200mm annual rainfall. This differs from the semi-arid rangelands that cover the majority of Kenya [6].

The virus is transmitted between animals and from animals to people by mosquitoes, however the most common route of infection for people during epidemics is exposure to infected animals or their products, particularly abortion material when affected animals are shedding large amounts of virus [7,8]. Slaughterhouse workers are at risk of exposure to infected materials such as blood through cutting animals’ throats and handling animal parts [8–10].

Most people infected by RVFV suffer mild or subclinical disease, although a small percentage will suffer severe disease. Fever, nausea, and vomiting are the most commonly reported clinical signs in people [11,12]. Other signs include large joint arthralgia, diarrhea, jaundice, right upper quadrant pain, and backache [11–13]. Ocular manifestations, including uveitis and retinitis, occur in 1.5–3% of patients and can result in permanent vision loss [8,12]. Severe forms of the disease occur in up to 10% of patients and include a haemorrhagic form that is associated with high fatality and a meningoencephalitis form manifested by neurological symptoms which may continue after resolution of the infection [8,14,15].

Clinical diagnosis of RVF may be hindered because of the similar presentation to other endemic mosquito-borne illnesses, such as malaria or dengue [11,16]. Diagnosis of RVF is made by virus isolation or polymerase chain reaction (PCR) in the early stage of clinical disease [17]. Virus neutralisation assays are the gold standard of antibody detection, but the requirement for live virus makes their use limited [18]. Enzyme-linked immunosorbent assays (ELISA) for Immunoglobulin M and IgG can be used for diagnosis and surveillance of RVF, identifying recent and historic exposure, respectively [19].

This study aimed to determine the seroprevalence of RVF antibodies in a community in western Kenya where disease outbreaks have not previously been reported, but where a prior study documented RVFV circulation [4]. A concurrent study in slaughterhouse workers aimed to determine if there was an occupational risk of RVFV seropositivity in an area that has not experienced RVF outbreaks similar to reports in areas that experience epizootics [8].

## Methods

### Ethical approval

Ethical approvals for the ‘People, Animals and their Zoonoses’ (PAZ) project community and slaughterhouse worker studies were granted by the Kenya Medical Research Institute (KEMRI) Ethical Review Committee (SCC Protocols 1701 and 2086, respectively). Written, informed consent was obtained from all participants; for children between 5 and 17 a parent or legal guardian provided consent. Consent forms were in English and Kiswahili.

### Study site

**Fig 1:**
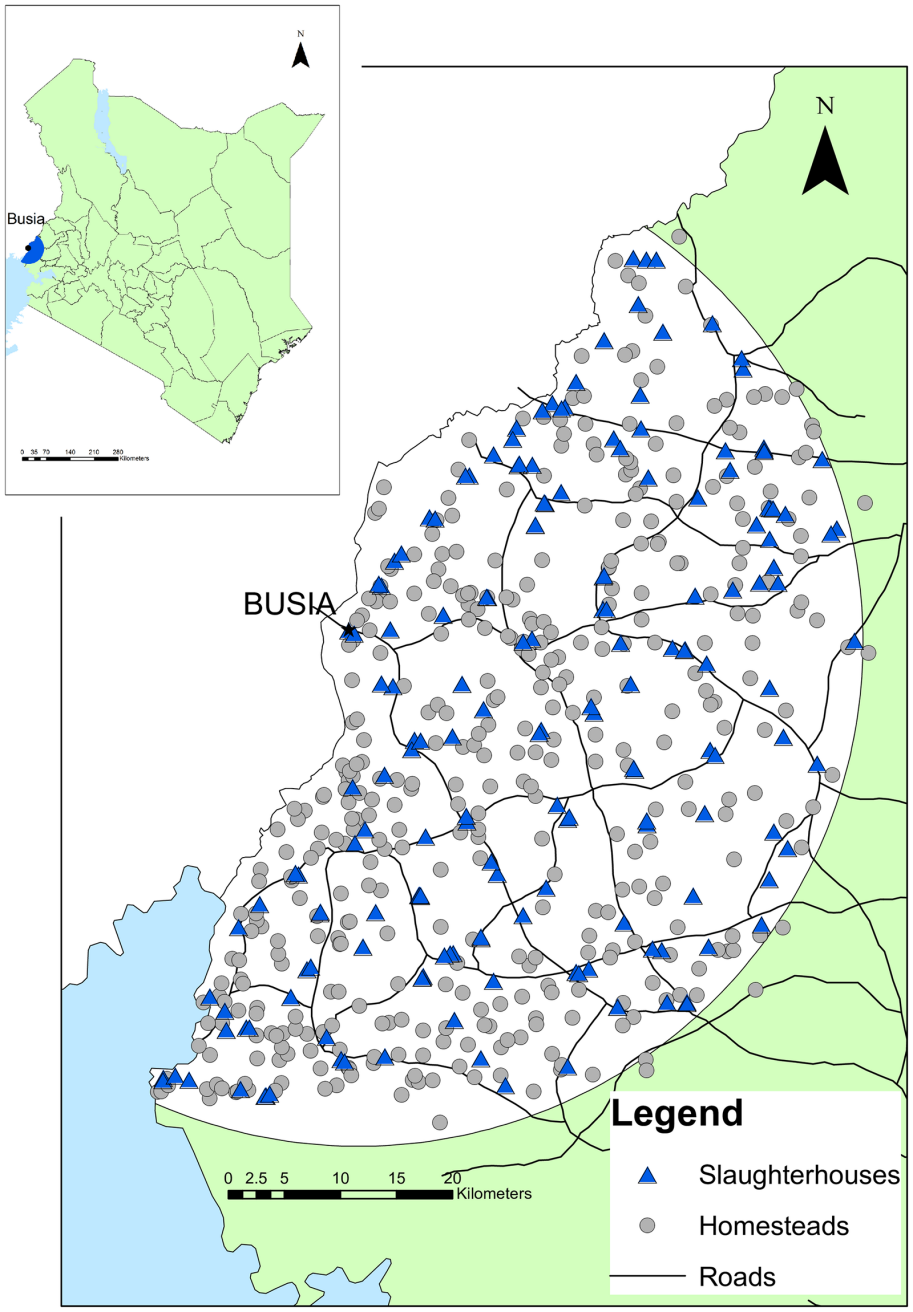
Map of the study area, indicating the distribution homesteads and slaughterhouses.

The study was conducted in western Kenya in the Lake Victoria Basin region on the border with Uganda (Fig 1). The study area was within a 45-kilometre radius from Busia town where the project laboratory was located (Fig 1). The region is predominantly rural but has a high population density with approximately 500 people per square kilometre (estimated from the Kenyan Human Population Census of 2009). The predominant ethnic groups are Luhya, Luo, and Teso. It is estimated that more than 40% of homesteads are below the poverty line [20]. The mean homestead size is 5 persons (estimated from the Kenyan Human Population Census of 2009). Mixed subsistence farming is the predominant source of livelihood for 75.6% of homesteads [21].

### Sampling frame

#### Community

The data used for this analysis incorporates information from the PAZ project. The PAZ project was a cross sectional serological study investigating people and animals for zoonoses and associated risk factors conducted between July 2010 and July 2012 in western Kenya [22].

Homesteads were randomly selected using a two-stage cluster design. The study area was divided into 143 sub-locations, which is the smallest administrative unit in Kenya. The number of homesteads selected from each sub-location was proportional to the cattle density i.e. more homesteads were sampled in sub-locations with more livestock. The information on livestock population density was obtained from the Divisional Livestock Production Office (DPLO) and based upon a 2005 livestock census inflated by 10% per year. The cattle population of the study site was estimated to be 557,418 cattle. The number of homesteads sampled per sub-location varied from 1 to 8. The human sample size was the number of individuals living in the selected homesteads.

A random set of points was generated within each sub-location using ArcMap version 9.1. The International Livestock Research Institute (ILRI) geographical information systems unit (http://www.ilri.org/gis/) provided shapefiles. A handheld Global Positioning System (GPS Garmin eTrex), was used in the field to locate each point. The nearest homestead within 300 metres of the point was recruited into the study. If there were no homesteads in the area or the homestead head refused to participate then a backup point was used. The homestead head was advised of the study aims and objectives and recruited into the study, and an appointment made for data collection and sampling the following week.

All homestead members aged over 5 years and not in third trimester pregnancy were invited to participate. Each participant was individually interviewed with a structured questionnaire that included questions regarding demographic data, health, and risk factors for zoonotic disease exposure. Age was collected in 5-year categories from 5 years of age. For the purposes of analysis these categories were recoded to make a continuous variable. The homestead head was asked a homestead-level questionnaire regarding animal ownership, wealth indicators, water source, and access to healthcare.

### Slaughterhouses

The study population included every ruminant slaughterhouse worker in the study area. The location of slaughterhouses in the study area was obtained from the former District Veterinary Officers (now County Directors of Veterinary Services) who had oversight over meat inspection (Fig 1). There were 88 ruminant slaughterhouses identified in the study area. Inclusion criteria specified all workers, aged over 18 years and present at the slaughterhouse on the day of sampling. Due to the time required to process the samples on the day of collection, the number of workers recruited from each slaughterhouse was limited to 12. The mean number of workers in the slaughterhouses was 9 and the mean number of animals slaughtered per week was 21. In slaughterhouses with 12 workers or less, all willing participants were recruited. In slaughterhouses with greater than 12 workers, a random selection of 12 willing participants from the workers present on the day was sampled. On the day of sampling workers were assigned a number. This was written on a piece of paper and placed in a container. Numbers were selected from the container until twelve participants were chosen.

A clinical officer from the project team, responsible for all medical examinations, could exclude participants for any underlying health condition where participation might affect them adversely, including third trimester pregnancy, under the age of eighteen, severe inebriation, aggression toward the project staff, and extreme old age (over 85 years).

This project investigated the current practices in slaughterhouses in western Kenya using two tools: 1) the foreman was asked questions related to the facilities and practices within the slaughterhouses as a unit; 2) individual workers were asked questions regarding knowledge, attitudes, hygiene practices, and health of the worker.

Questionnaire data were recorded in a Palm operating system (Palm OS) personal digital assistant (PDA) using Pendragon Forms 5.1 (Pendragon Software Corporation, Libertyville, IL, USA). Microsoft Access databases were used to manage data.

### Biological sample collection

Samples were collected from every participant who gave informed consent. A clinical officer collected 10mls of blood from each participant (10ml plain Becton, Dickinson and Company (BD) Vacutainer) using a 21G or 23G BD Vacutainer Safetylok blood collection set and sterile technique.

### Laboratory analysis

Sera from study participants were tested by indirect ELISA for presence of anti-RVFV IgG antibodies at Stanford University School of Medicine, Stanford, CA, as described previously [4,5,23]. Seropositive or seronegative results were compared to plaque reduction neutralization test (PRNT)-confirmed positive and negative controls for RVFV. The cut-off values used to determine positive readings were calculated by dividing the average positive control optical density (OD) value on each plate by two. Cut-off values used to determine negative readings were calculated by multiplying the average negative control OD value on each plate by two.

### Prevalence estimation

Confidence intervals (CI) around apparent prevalence estimates were calculated using the *epi*.*prev* function in the *EpiR* package [24] of the R environment for statistical computing, version 3.0.2 (http://cran.r-project.org/). To account for the hierarchical nature of surveys in both the community and in slaughterhouse workers [25], design-based adjustment was implemented using the *svydesign* procedure in the *Survey* package in R [26]. Sampling weights were calculated for the community sample by dividing the number of people per division (from the Kenyan Human Population Census of 2009) by the number of people sampled in each division. Sampling weights for slaughterhouse were calculated by dividing the total number of workers by the number sampled in each slaughterhouse. Homestead and slaughterhouse were included as clustering variables in the community and slaughterhouse samples, respectively. The true prevalence estimate accounting for the RVFV IgG ELISA sensitivity and specificity, but without accounting for the complex survey design, were calculated using the *truePrev* function in the *prevalence* package [27] of R. The sensitivity and specificity of the test have been reported to be 100% and 95.3–100% respectively [4,5].

### Logistic regression model

Multi-level logistic regression models were used to identify risk factors for RVFV seropositivity in community members and in slaughterhouse workers and estimate the strength of the relationship with the outcome. A multi-level mixed effects logistic regression model was used to account for the clustering of individuals within homesteads. A separate multi-level mixed effects logistic regression model was used to account for the clustering of workers within slaughterhouses. Univariable logistic regression was used to screen variables of interest, against disease seropositivity at the individual level. Variables were included from both the individual and homestead/slaughterhouse level. Variables screened were those that have been previously identified as risk factors associated with RVFV seropositivity for community members and slaughterhouse workers (S1 Table). Multi-level logistic regression models were developed using *glmer* function in the *lme4* package [28].

Group level variation in the final models was examined to assess the importance of the homestead/slaughterhouse in explaining individual risk of RVFV seropositivity. The Median Odds Ratio (MOR) was calculated for the final models. The MOR expresses the between group variance on the odds ratio scale, and therefore provides a measure of the between group variability in individual risk for an outcome that can be interpreted on the same scale that risk factors are interpreted [29,30]. The MOR is estimated using Eq 1 [30].

$$MOR=exp\sqrt{2xV_{A}}x\;0.6745$$ (1)

The intraclass correlation coefficient (ICC) was calculated for the final model. The ICC represents correlation in the probability of seropositivity at the homestead/slaughterhouse level. It was estimated using the latent variable method using Eq 2 [30].

$$ICC=\;\frac{V_{A}}{V_{A}+\;\frac{\pi^{2}}{3}}$$ (2)

### Model diagnostics

Variance Inflation Factors (VIFS) were calculated to check for collinearity. VIFS >4 were considered a problem and the variable removed from the model. The Moran’s I statistic was calculated to check for spatial autocorrelation in homestead/slaughterhouse level residuals which can influence the stability of model co-efficients. The Moran’s I statistic measures if the outcome (group level residual log odds of seropositivity) is clustered or randomly distributed through space [31]. The Moran’s I statistic was calculated using the *ape* package [32] in R. A histogram of the group-level residuals was made to check for normality.

### Mapping

Homesteads and slaughterhouses were georeferenced using a handheld GPS device (Garmin eTrex). The locations were mapped using ArcGIS version 9.1 and version 10.2.2 (ESRI, Redlands, CA, USA).

For mapping purposes, homesteads and slaughterhouse were considered positive if one or more inhabitants/workers were seropositive for RVFV. The spatial scan statistic was used to determine if there was any evidence of clustering of the RVFV seropositive homesteads [33]. A Bernoulli model was used with 999 iterations in SatScan version 9.0 (www.satscan.org).

A kernel smoothing approach was used to map the intensity of positive homesteads and slaughterhouses using the *sparr* [34] package in R with a fixed bandwidth of 5km and correction for edge effects. A bandwidth of 5km was chosen because it is the approximate diameter of sublocations in the study area. The kernel intensity of seropositive homesteads/slaughterhouses was divided by the kernel intensity of the all homesteads/slaughterhouses in the study area creating a “risk” surface. This technique is not a test for clustering but produces spatially smooth risk maps that allow areas with the greatest risk for seropositivity to be identified.

## Results

### Community

A total of 1,861 individuals were sampled in 384 homesteads. Participating individuals were aged between 5 and 85 years with 969 (52%) of participants aged below 20 years. Seventy-four percent of participants reported owning livestock including cattle (62%); sheep (18%) and goats (33%).

Fifteen people were seropositive for RVFV hence the seroprevalence in the community was 0.8% (95% CI 0.5–1.3%). The survey-adjusted seroprevalence was 0.5% (95% CI 0.2–0.8%). The true prevalence accounting for the sensitivity and specificity of the diagnostic test was 0.1% (95% CI 0.0–0.2%).

Using univariable logistic regression there was not a significant difference in seropositivity between genders (Table 1). There was a significant difference across age with only one seropositive individual in the 5–19 year age group giving a seroprevalence of 0.1% (95% CI 0.0–0.6%); compared with 14 positives (1.6%, 95% CI 0.9–2.6%) in the over 20 year age group. The youngest seropositive participant was aged between 10–14 years (age data was collected in categories).

**Table 1:** Results of univariable analysis for risk factors for RVFV seropositivity in community participants.

Variable	Number (%) n = 1861	RVFV positive (%)	OR (95% CI)	*p* value
**Male**	856 (46.0)	4 (0.5)	0.4(0.1–1.3)	0.143
**Female**	1005 (54.0)	11 (1.1)		
**Age (5 year intervals)**			1.2 (1.1–1.4)	<0.001
**Individual risk factors**				
Drinking animal blood*	369 (19.9)	6 (1.6)	2.7 (1.0–7.6)	0.061
	1482 (80.1)	9 (0.6)		
Slaughtering	299 (16.1)	5 (1.7)	2.6 (0.9–7.8)	0.079
	1557 (83.9)	10 (0.6)		
Farmer	616 (35.7)	9 (1.5)	2.7 (1.0–7.7)	0.058
	1111 (64.3)	6 (0.5)		
Handling animal abortions	32 (1.7)	1 (3.1)	4.2(0.5–32.7)	0.174
	1824 (98.3)	14 (0.8)		
Animal birthing	141 (7.6)	2 (1.4)	1.9 (0.4–8.4)	0.408
	1715 (92.4)	13 (0.8)		
Skinning	66 (3.6)	1 (1.5)	2.0 (0.3–15.1)	0.521
	1790 (96.4)	14 (0.8)		
Cattle shelter in house	64 (3.4)	0 (0)	NA	NA
	1797 (96.6)	15 (0.8)		
Goats/sheep shelter in house	58 (3.1)	2 (3.4)	4.9 (1.1–22.3)	0.039
	1803 (96.9)	13 (0.7)		
**Livestock ownership (homestead)**				
Cattle	1162 (62.4)	10 (0.9)	1.2(0.4–3.5)	0.735
	699 (37.6)	5 (0.7)		
Sheep	342 (18.4)	5 (1.5)	2.2(0.8–6.6)	0.144
	1519 (81.6)	10 (0.7)		
Goats	612 (32.9)	9 (1.5)	3.1(1.1–8.7)	0.033
	1249 (67.1)	6 (0.5)		
**Homestead risk factors**				
Abortion in the herd	173 (9.3)	2 (1.2)	1.5 (0.3–6.7)	0.591
	1688 (90.7)	13 (0.8)		
Slaughter cattle at home	175 (9.4)	4 (2.3)	3.6 (1.1–11.5)	0.029
	1686 (90.6)	11 (0.7)		
Slaughter goat/sheep at home	128 (6.9)	4 (3.1)	3.4 (1.0–12.4)	0.058
	1733 (93.1)	11 (0.6)		

* Blood was consumed raw and also cooked.

Variables that have been previously described as being associated with RVF seropositivity were tested using univariable logistic regression analysis (Table 1). Variables that were significantly associated with RVFV seropositivity included: increasing age (OR 1.2; 95% CI 1.1–1.4, p < 0.001), owning goats (OR 3.1; 95% CI 1.1–8.7, p = 0.033); slaughtering cattle at the homestead (OR 3.6; 95% CI 1.1–11.5, p = 0.029) and sheltering goats and sheep in the house (OR 4.9; 1.1–22.3, p = 0.039). Other variables that have been associated with RVFV seropositivity in previous studies such as handling animal abortus, and assisting with animal birthing were positively associated with RVFV seropositivity but not significantly using the traditional level of 0.05 (Table 1).

The small number of positive results in the community sample (n = 15) limited the inclusion of all significant univariable effects in a multivariable model [35]. Instead, we focused only the effect of slaughtering animals, with control for the potential confounding effect of age. No further model selection was performed. Increasing age continued to predict seropositivity (OR 1.2 95% CI 1.1–1.4, p <0.001) and there was evidence of the positive effect of slaughtering cattle (OR 3.3; 95% CI 1.0–10.5, p = 0.047) (Table 2).

**Table 2:** Results of multi-level analysis for risk factors for RVFV seropositivity in the community.

Variable	OR (95% CI)	*p* value	VIFs
**Individual factors**			
Age	1.2 (1.1–1.4)	<0.001	1.001
**Homestead level factors**			
Slaughter cattle at home	3.3 (1.0–10.5)	0.047	1.001

The Moran’s I statistic demonstrated no evidence of residual spatial autocorrelation (value = 0.004, p-value = 0.417). The histogram of the group level residuals had a normal distribution. The MOR was 1 and ICC less than 1%, indicating that very little of the variation in individual risk of seropositivity from the final model was associated with the factors operating at the homestead level.

**Fig 2:**
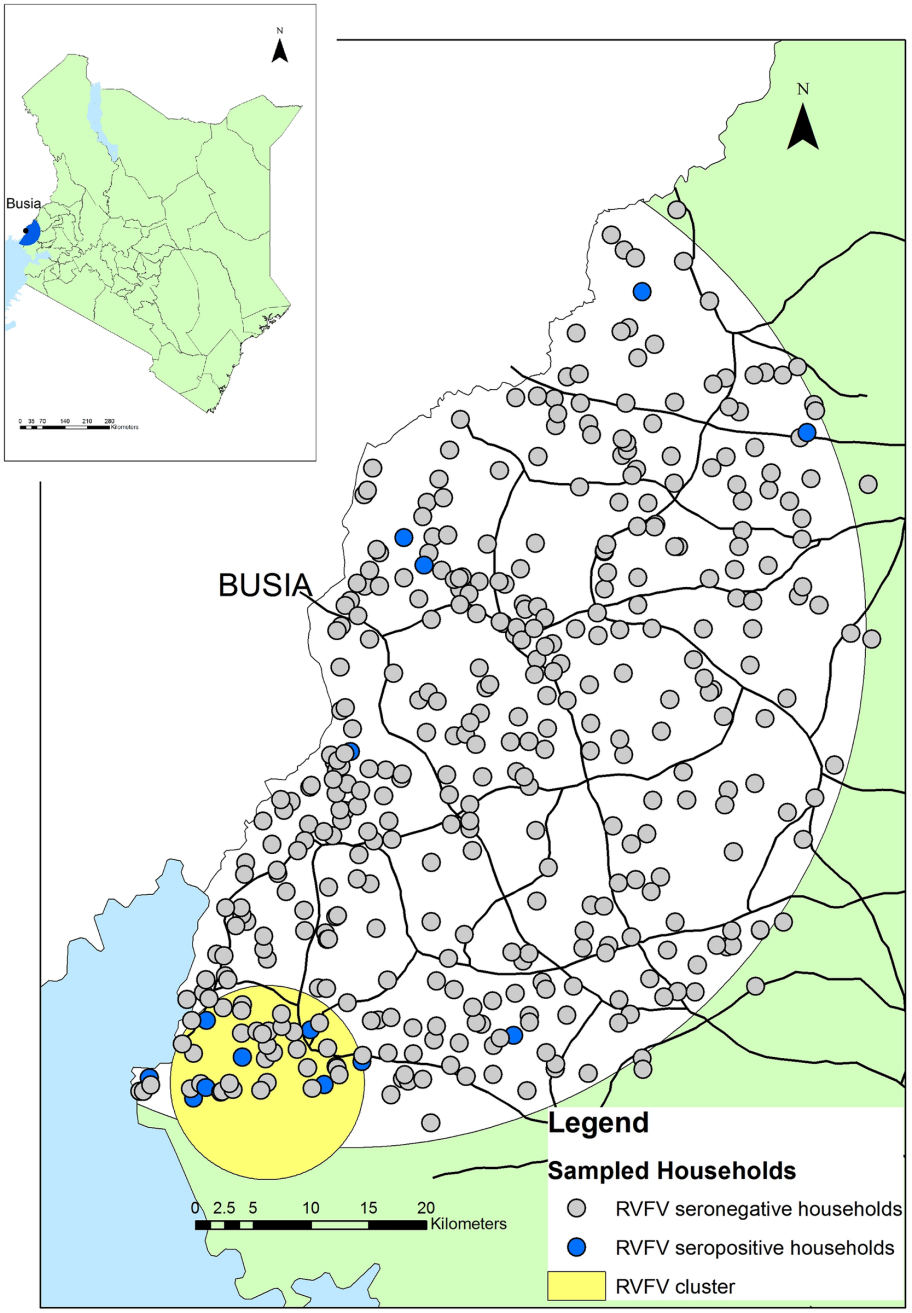
Map of the distribution of RVFV seropositive and seronegative homesteads and the statistically significant (p<0.05) cluster of elevated relative risk for homestead level RVFV seropositivity.

**Fig 3:**
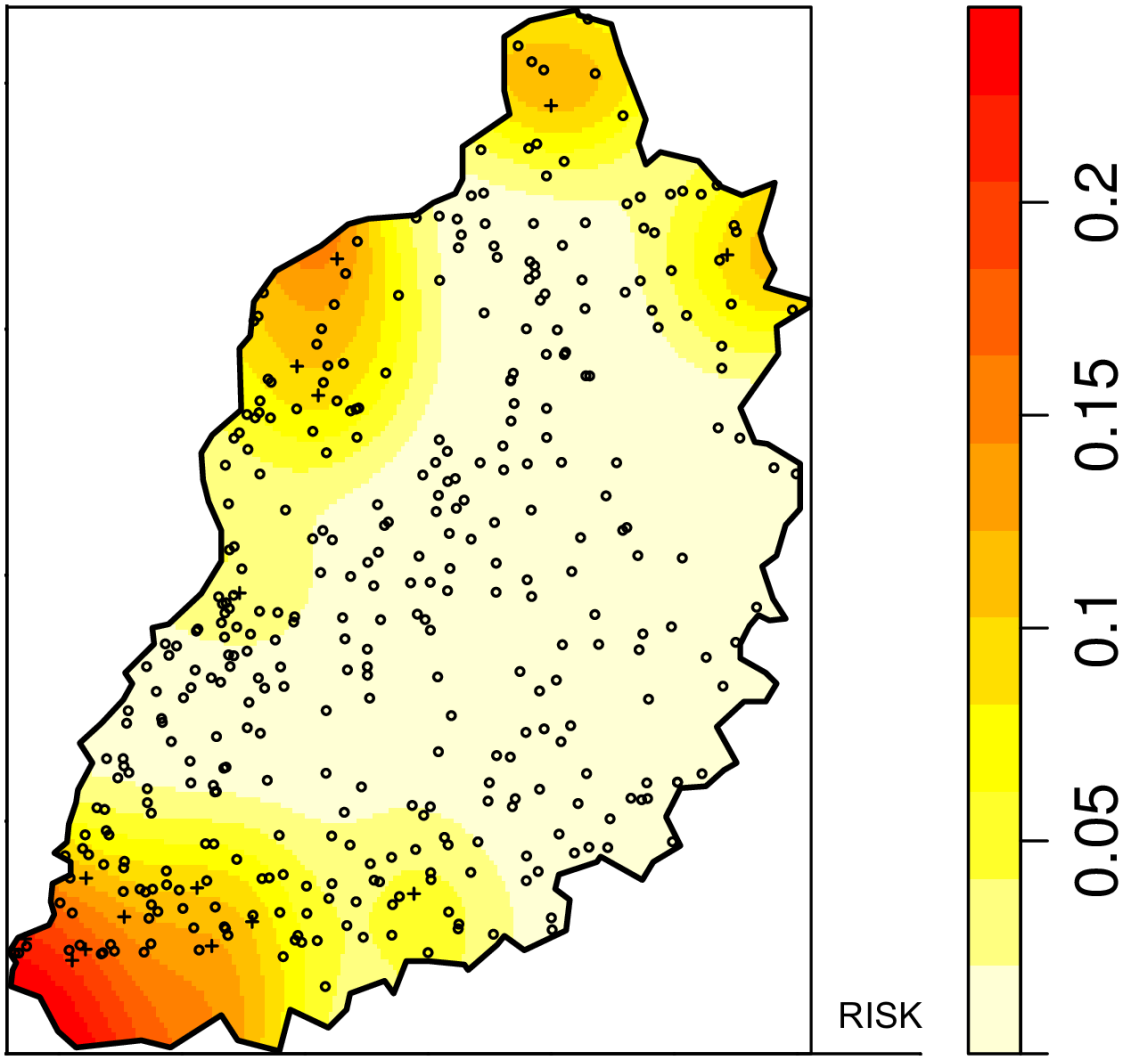
Spatially-smoothed risk map for RVFV seropositivity in the community sample. The points are the locations of the homesteads.

A significant spatial cluster was detected in the south of the study area. The relative risk (RR) of homesteads inside the cluster compared to outside was 45.57 (p-value = <0.001) (Fig 2). The results of the kernel density mapping for RVFV in homesteads (Fig 3) suggest the greatest risk for RVFV seropositivity in the community was to be near Lake Victoria in the southwest of the study area.

### Slaughterhouse

A total of 553 slaughterhouse workers were sampled in 84 ruminant slaughterhouses. Four slaughterhouses refused to participate in the study. The majority of slaughterhouse workers were men (96.8%). The age of the slaughterhouse workers ranged from 18–82 years with a median age of 38 years. The roles in the slaughterhouse included flayers (74.7%), slaughtermen (11.6%), and cleaners/foremen (13.7%). There were 18 female slaughterhouse workers and the role of women within the slaughterhouse differed to men with only one female flayer (5.6%) and the remainder were cleaners (94.4%).

The number of workers seropositive for RVFV was 14 giving an apparent seroprevalence of RVFV in slaughterhouse workers of 2.5% (95% CI 1.5–4.2). The survey-adjusted prevalence was 2.6% (95% CI 1.3–3.9). The true prevalence accounting for the sensitivity and specificity of the test was 0.3% (95% CI 0.0–1.2%).

Of the 18 female slaughterhouse workers, none were seropositive for RVFV (Table 3). Using univariable logistic regression the only variable associated with RVFV seropositivity in slaughterhouse workers was being the slaughterman (OR 3.2 95% CI 1.0–10.5, p = 0.055).

**Table 3:** Results of univariable analysis for risk factors for RVFV seropositivity in slaughterhouse workers.

Variable	Number (%) n = 553	RVFV positive (%)	OR (95% CI)	P value
**Male**	535 (96.7)	14 (2.5)	NA	
**Female**	18 (3.3)	0		
**Age (years)**			1.02 (0.98–1.05)	0.329
**Individual factors**				
**Animal contact outside work**				
Cattle	406 (73.4)	11 (2.7)	1.3 (0.4–4.8)	0.665
	147 (26.6)	3 (2.0)		
Sheep	152 (27.4)	4 (2.6)	1.1 (0.3–3.4)	0.920
	401 (72.6)	10 (2.5)		
Goats	239 (43.2)	4 (1.7)	0.5 (0.2–1.7)	0.270
	314 (56.8)	10 (3.2)		
**Time as slaughterhouse worker (years)**			0.99 (0.94–1.05)	0.775
**Number of animals slaughtered by worker per week**			0.99 (0.89–1.09)	0.804
**Animals slaughtered by workers**				
Cattle only	361 (65.2)	12 (3.3)	3.3 (0.7–14.8)	0.123
Cattle, goats and sheep	192 (34.8)	2 (1.0)	Ref	
**Job in the slaughterhouse**				
Slaughterman	64 (11.6)	4 (6.3)	3.2 (1.0–10.5)	0.055
Other jobs	489 (88.4)	10 (2.0)		
**Lived outside the study area**				
Yes	182 (33.0)	6 (3.3)	1.5 (0.5–4.5)	0.429
No	371 (67.0)	8 (2.2)		
**Slaughterhouse factors**				
**Number of animals slaughtered in facility per week**			0.99 (0.95–1.02)	0.421
**Animal type slaughtered**				
Cattle only	283 (51.2)	9 (3.2)	1.8 (0.6–5.3)	0.319
Cattle, goats and sheep	270 (48.8)	5 (1.8)		

Due to the small number of positive samples, only three variables were included in the final multilevel model for RVFV seropositivity in slaughterhouse workers [35]. These were variables from the univariable analysis that had been previously reported as high risk for RVFV seropositivity and had p<0.2. Age was included in the model since it is a common confounder [5]. No further model selection was performed. The final model included age, if the worker only slaughtered cattle and being the slaughter man (Table 4). Being the slaughter man was significantly associated with RVFV seropositivity in slaughterhouse workers after multi-level analysis (OR 3.5; 95% CI 1.0–12.1, p = 0.047).

**Table 4:** Results of multi-level analysis for risk factors for RVFV seropositivity in slaughterhouse workers.

Variable	OR (95% CI)	*p* value	VIFs
**Individual factors**			
Age	1.01 (0.98–1.05)	0.511	1.017
Slaughterman	3.5 (1.0–12.1)	0.047	1.066
Worker slaughtered only cattle	3.8 (0.8–17.5)	0.085	1.049

The histogram of the group level residuals had a normal distribution. The MOR was 1 and ICC less than 1%, indicating that very little of the variance is associated with factors operating at the level of the slaughterhouse and most of the variation is at the individual level.

**Fig 4:**
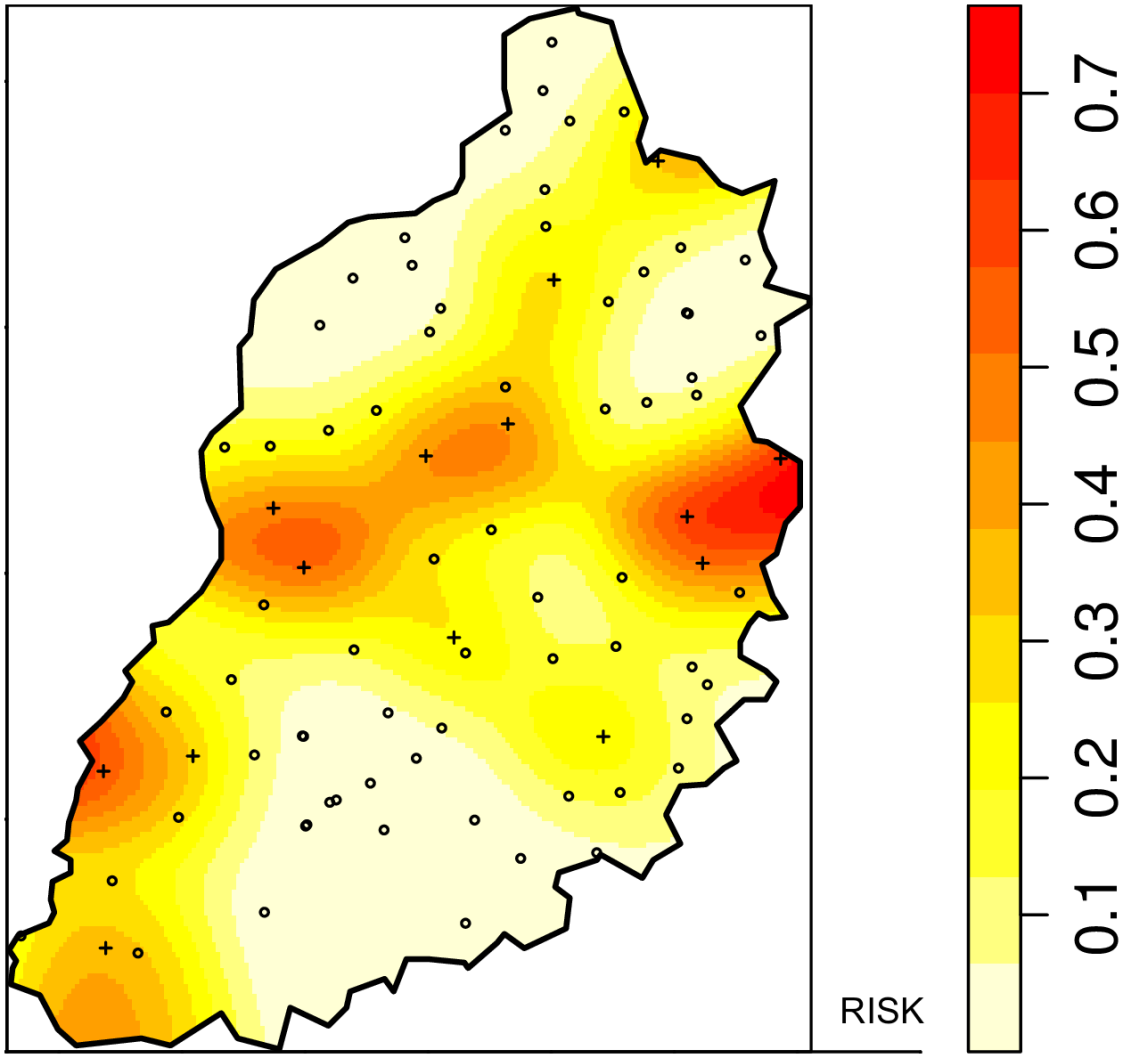
Spatially-smoothed risk map for RVFV seropositivity in slaughterhouse workers. The points are the locations of the individual slaughterhouses.

The kernel density mapping for RVFV in slaughterhouses (Fig 4) showed the areas of greatest risk for RVFV seropositivity in slaughterhouse workers to be through the center of the study area and along the border with Uganda.

## Discussion

There have been two Rift Valley Fever epidemics in East Africa in the past 20 years [3]. In both outbreaks there were no human cases recorded in western Kenya. Research efforts focusing on the inter-epidemic transmission of RVFV have reported the seroprevalence in people in high-risk areas to range from 6% (95% CI 2.7–11.8) to 20% (95% CI 14.0–29.2) [5]. In locations believed unaffected by RVFV outbreaks, the seroprevalence estimates range from 0% (95% CI 0–3.03%) to 3% (95% CI 0.94–6.78%) [4]. The apparent seroprevalence for RVFV in the community in this study was 0.8% (95% CI 0.5–1.3). This is consistent with a previous report from Kabobo in the western highlands of Kenya (1%; 95% CI 0.03–5.45) [4]. The study area presented here is a semi-humid environment with high average annual rainfall (>1200 mm per annum) similar to that of Kabobo [6]. LaBeaud et al (2007) theorised that the different climatic conditions across regions would account for the difference in seroprevalence estimates [4]. Semi-arid regions with seasonal flooding events allow annual RVFV transmission resulting in high seroprevalence in contrast to semi-humid climates where only extensive rainfall allow RVFV transmission and hence low seropositivity [4].

The risk map demonstrated the highest risk for RVFV seropositivity in the community is in the south west of the study area bordering Lake Victoria. In addition there was a significant clustering of seropositive homesteads in this area (p <0.001). Previous studies have demonstrated an association between RVFV seropositivity in ruminants and proximity to water bodies [36,37] and RVFV outbreaks have been associated with flooding and the resulting increased vectors [38,39]. The southern area of the study site is the Nzoia and Yala rivers wetland zone which periodically floods in heavy rains [40]. The area around Lake Victoria is likely to be highly suitable for mosquito breeding that might allow for inter-epidemic transmission of RVFV from vectors.

RVFV seropositivity in the community examined in this study was associated with increasing age (OR 1.2, 95% CI 1.1–1.4, p<0.001). This is consistent with findings by other researchers investigating risk factors for RVFV [5,41]. As concluded by LaBeaud et al (2015) [42] this is potentially the result of older people having more time to be exposed to infected materials and vectors since IgG antibodies for RVFV are considered to be lifelong [43]. In addition, young people are potentially less likely to be involved in risk practices such as handling livestock abortions and slaughtering [42]. The youngest seropositive individual was between 10–14 years (more accurate age data was not collected) and was potentially exposed to RVFV between 1998 and 2012. There was a countrywide outbreak in 2006–2007, however western Kenya was considered free of the disease at that time [44]. The low seroprevalence for RVFV in this study may indicate that there has not been a large amount of endemic RVFV circulation in this area in the past 20 years.

Slaughtering cattle at the homestead was demonstrated to be a risk factor for RVFV seropositivity (OR 3.3; 95% CI 1.0–10.5, p = 0.047). This is similar to previous studies that have demonstrated slaughtering animals and handling animals parts are risk factors for RVFV seropositivity [8,10,45]. Slaughtering animals during an RVFV outbreak has been reported to be a risk factor for severe disease and death [7]. The public health response to RVFV outbreaks in the past has been to ban slaughtering to reduce the human cases [7,9]. However, this results in significant economic losses to producers and other stakeholders in the value chain [46]. This may result in movement of infected animals away from the outbreak area spreading the virus to unaffected regions [9]. It is possible that during the last epidemic animals were moved into the study areas from affected areas. Movement of infected livestock is believed to have spread RVFV to the Arabian peninsula and Egypt [47].

The apparent seroprevalence of RVFV in slaughterhouse workers was 2.5% (95%CI 1.5–4.2). These results are comparable to other studies that have been conducted in RVFV endemic areas. For example, in Egypt and Saudi Arabia, RVFV seroprevalence in slaughterhouse workers was 2% and 0.72% respectively [10,48]. The seroprevalence of RVFV antibodies in slaughterhouse workers (2.5%) was higher than in the community population over 20 years old (1.6%), supporting the hypothesis that slaughterhouse workers are at higher risk for exposure to RVFV and that they may act as sentinels for RVFV, even in areas with low transmission [49]. The area with the highest spatial risk is in slaughterhouses located in the central region of the study area, along the main road networks into the study area, further indicating the possibility that animal cases may be imported from areas outside the study area. This highlights a difference in the risk profile between the community and slaughterhouse workers in this area suggesting that slaughterhouse workers risk is related to the movement of animals and not proximity to water. This hypothesis requires further investigation.

The slaughterman who is directly responsible for slitting the animal’s throat is previously reported to be at risk for RVFV seropositivity in regions where epizootics occur [8,10]. It is likely that aerosolization of blood at slaughter is a means for transmission of RVFV [50]. The odds of being RVFV seropositive was significantly higher in slaughtermen (OR 3.5, 95% CI 1.0–12.1, p = 0.047) compared to other roles in the slaughterhouse demonstrating that this is a high-risk position. Animals with RVF can present with abortion, hemorrhage, dyspnea, coughing, bloody discharges, anorexia, weakness [44] and it is possible that they might be removed from slaughter through antemortem inspection in order to protect workers from exposure. The impact of antemortem inspection on reducing RVFV exposure could not be determined from this study but should be considered for future investigations.

The ELISA conducted in this study has been used in previous studies [4,5,23]. The sensitivity has been described to be 100% where confirmatory testing was carried out and the reported specificity ranged between 95.3–100% [4,5]. It was not possible to perform plaque neutralization confirmatory testing in this study. The true prevalence rates in both the community and slaughterhouse sample were substantially reduced when accounting for the vagaries of the diagnostic test, with the 95% confidence interval including zero. Genus-specific cross-reactivity is a known limitation with ELISAs. Although ELISAs are effective for general surveillance, the sensitivity and specificity are less than those for PRNT [5,23,41]. Participant exposure to other bunyaviruses, may have elicited a cross-reactive seropositive result, despite using antigenic proteins specifically derived from RVFV as a coating antigen. As a result, a fraction of our subjects may have been misclassified in terms of RVFV exposure. PRNTs were not performed to determine the species-specific origin of the IgG antibodies detected by ELISA, yet serum pools derived from samples confirmed as seropositive and seronegative for anti-RVFV IgG by PRNT were used as controls. Given the low risk of exposure to other *Bunyaviridae* and phlebovirsuses [51,52], in the Busia region, we are confident that the results from this study are representative of RVFV exposure only.

Our ability to explore a range of predictors of seropositivity was limited by the small numbers of RVFV seropositive individuals. The self reported questionnaire was focused on risk factors for zoonotic disease associated with animal contact and did not include questions related to mosquito exposure and clinical data relevant to RVFV. In addition recall bias may have influenced responses regarding exposures. Conclusions cannot be made about when and where the individuals encountered the virus since antibodies to RVFV are likely to be life-long [43]. It is possible that the seropositive people were infected with RVFV during travel outside the study area. It was not possible to test this hypothesis with the data available from this study. However, this is unlikely considering the clustered nature of the community sample and the plausibility of the identified risk factors.

## Conclusion

This study investigated the epidemiology of RVFV in people in western Kenya. The study area was distinctly different from the regions of Kenya where RVFV outbreaks have been reported and where previous investigations have shown inter-epidemic transmission of RVFV. This study reported a low seroprevalence in the community and highlighted several previously identified risk factors in people including contact with animals and animal products. The study demonstrated that slaughterhouse workers are at a higher risk for exposure to RVFV and might be a sentinel for disease emergence. Methods to control infected animals being slaughtered such as antemortem meat inspection and education of workers should be implemented. The results suggest that RVFV virus is circulating in western Kenya. Improved surveillance in low risk areas is recommended particularly during countrywide outbreaks, to more accurately determine RVF burden.

## Supporting information

S1 Table List of individual and homestead/slaughterhouse level covariates used for analysis of human seropositivity to RVFV. (DOCX)

S2 Table STROBE checklist. (DOC)
