# Supplementary material for: The sero-epidemiology of Rift Valley fever in people in the Lake Victoria Basin of western Kenya
Source: PLoS Negl Trop Dis. 2017 Jul 7;11(7):e0005731. doi: 10.1371/journal.pntd.0005731 (PMC5517073; doi:10.1371/journal.pntd.0005731)
Supplement: S1 Table — (DOCX) [file pntd.0005731.s001.docx]

| Community variables | Slaughterhouse variables |
| --- | --- |
| Individual risk factors | **Individual risk factors** |
| Gender | Gender |
| Age (years) | Age (years) |
| Drinking animal blood* | **Animal contact outside work** |
| Slaughtering | Cattle |
| Farmer | Sheep |
| Handling animal abortus | Goats |
| Animal birthing | Time as a slaughterhouse workers (years) |
| Skinning | Number of animals slaughtered / week |
| Cattle shelter in house | **Species of animals slaughtered by worker** |
| Goats/sheep shelter in house | Cattle only |
| Livestock ownership | Cattle, goats and sheep |
| Cattle | **Job in the slaughterhouse** |
| Sheep | Slaughterman |
| Goats | Other jobs |
| Household risk factors | Lived in another area |
| Abortion in the herd | **Slaughterhouse factors** |
| Cattle slaughter | **Animals type slaughtered** |
| Goat/sheep slaughter | Cattle only |
|  | Cattle, goats, sheep |
